# An open access medical knowledge base for community driven diagnostic decision support system development

**DOI:** 10.1186/s12911-019-0804-1

**Published:** 2019-04-27

**Authors:** Lars Müller, Rashmi Gangadharaiah, Simone C. Klein, James Perry, Greg Bernstein, David Nurkse, Dustin Wailes, Rishi Graham, Robert El-Kareh, Sanjay Mehta, Staal A. Vinterbo, Eliah Aronoff-Spencer

**Affiliations:** 1 Design Lab, UCSD, San Diego, CA USA; 2 Amazon Web Services, Palo Alto, CA USA; 3 Division of Infectious Diseases, UCSD, San Diego, CA USA; 4 Division of Biomedical Informatics, UCSD, San Diego, CA USA; 5 School of Medicine, UCSD, San Diego, CA USA; 6 Department of Information Security and Communication Technology, Norwegian University of Science and Technology, Trondheim, Norway; 7 Division of Hospital Medicine, UCSD, San Diego, CA USA

**Keywords:** Decision support systems, clinical (D020000), Diagnosis, differential (D003937), Knowledge bases (D051188)

## Abstract

**Introduction:**

While early diagnostic decision support systems were built around knowledge bases, more recent systems employ machine learning to consume large amounts of health data. We argue curated knowledge bases will remain an important component of future diagnostic decision support systems by providing ground truth and facilitating explainable human-computer interaction, but that prototype development is hampered by the lack of freely available computable knowledge bases.

**Methods:**

We constructed an open access knowledge base and evaluated its potential in the context of a prototype decision support system. We developed a modified set-covering algorithm to benchmark the performance of our knowledge base compared to existing platforms. Testing was based on case reports from selected literature and medical student preparatory material.

**Results:**

The knowledge base contains over 2000 ICD-10 coded diseases and 450 RX-Norm coded medications, with over 8000 unique observations encoded as SNOMED or LOINC semantic terms. Using 117 medical cases, we found the accuracy of the knowledge base and test algorithm to be comparable to established diagnostic tools such as Isabel and DXplain. Our prototype, as well as DXplain, showed the correct answer as “best suggestion” in 33% of the cases. While we identified shortcomings during development and evaluation, we found the knowledge base to be a promising platform for decision support systems.

**Conclusion:**

We built and successfully evaluated an open access knowledge base to facilitate the development of new medical diagnostic assistants. This knowledge base can be expanded and curated by users and serve as a starting point to facilitate new technology development and system improvement in many contexts.

**Electronic supplementary material:**

The online version of this article (10.1186/s12911-019-0804-1) contains supplementary material, which is available to authorized users.

## Background

Clinical decision making, a cornerstone of quality healthcare, has been and remains challenging [1]. The earliest attempts to integrate artificial intelligence (AI) into healthcare were diagnostic decision support systems (DDSS) [2–4]. DDSS support the diagnostic process by generating differential diagnoses from provided observations. The first DDSS were inspired by the reasoning of human experts and stored medical knowledge in structured knowledge bases. However, these systems failed to find wide acceptance [5–7]. Over the past decades, knowledge-based systems in AI have been replaced by machine learning (ML) platforms that learn from large amounts of data. Progress in ML in healthcare [8, 9] suggests that well-curated medical knowledge bases are no longer required and we can rely on analysis of existing medical textbooks, publications [10, 11] or large scale unstructured patient data. We argue ML methods and medical knowledge bases complement each other and that we lack open source diagnostic knowledge bases to integrate both approaches for new DDSS which combine their strengths.

The envisioned decision support systems would integrate ML-based AI, structured knowledge-based algorithms and heuristics similar to the dual system theory of human cognition [12] which distinguishes fast and non-conscious thinking (System 1) and analytical, slow and conscious (System 2) thinking. ML delivers pattern recognition that currently drives progress in image and voice recognition, but these advances don’t translate directly to DDSS as “each application requires years of focused research and a careful, unique construction [13]”. Knowledge-based systems [2–4] were inspired by the diagnostic methods taught in medical school, (e.g. Bayesian reasoning). The underlying knowledge base stores medical information in a structured manner so that a computer can automatically recommend diagnoses and a human can understand the differences in these choices. Not unlike human cognition, ML and knowledge-based systems have their strengths and weaknesses but likely perform best in combination.

The diagnostic process is an iterative process, progressing from an initial differential diagnosis based on prior probabilities of disease to diagnostic closure based on test results and progress of the disease. DDSS need to support this incremental nature of the diagnostic process [1] and challenge clinicians’ reasoning for each step; they cannot act as a “greek oracle” [14] and simply provide an answer once without explanation. Isabel [10] and FindZebra [11], for example, use text mining to search existing literature but cannot facilitate learning by explaining results or scaffold iterative queries and workup routines, functionality demonstrated by recent knowledge-based systems such as VisualDx [15]. ML excels in analyzing large amounts of data but the reasoning is not transparent. Recent approaches [16] provide some intuition about the overall function of the ML algorithm but cannot provide a deep understanding of a specific decision. Likewise, if only small amounts of data are available, knowledge-based systems can fill the gap. Knowledge bases can also complement machine learning approaches by explaining results generated by ML from a medical perspective.

New prototypes that aim to explore this design space cannot build on existing medical knowledge bases (KB). Medical ontologies and the UMLS metathesaurus [17] standardize the vocabulary but often not the required relationship between medical observations and explanations. Hence, designers of DDSS are forced to build their own knowledge bases and often end up with purely academic solutions [18–20]. Textbook knowledge is available in databases on the internet [21, 22], but the structured data most algorithms require has historically been stored in proprietary medical knowledge bases of the specific DDSS [2–4]. These knowledge bases are not accessible for building new DDSS, as they are either no longer maintained [2, 3] or part of a proprietary DDSS [4, 15, 23]. The design and curation are time-consuming and costly as they require specialized medical and technical knowledge [24].

In this paper we present an open access knowledge base to foster iterative improvement of diagnostic support and provide a basis for future systems that integrate ML and knowledge-based systems. A DDSS prototype, Doknosis, was developed to evaluate the knowledge base against well described commercial systems. For this report our evaluation is limited to a single algorithm and medical cases with single disease explanations.

## Construction and content

The curated KB holds medical diagnoses and medications (explanations) with associated observations recorded from primary literature and medical texts (such as [25–30]). The knowledge base was first developed and targeted for use by medical trainees in Mozambique and Sub-Saharan Africa [31]. Tropical Medicine and Infectious diseases were selected as the initial focuses of development and testing. The database, containing over 2000 unique diseases and nearly 450 medications at the time of this report, was then expanded to cover a broad range of illnesses ranging from medication side effects to both common and extremely rare medical diseases.

The KB data structure is inspired by the Bayesian Model of reasoning. This structure, essentially a key-value dictionary of estimated prior and conditional probabilities, is the substrate for algorithms developed to navigate the differential space and explore varied approaches for inferring and ranking possible diagnoses. The knowledge base was designed to be (a) machine readable and readily integrated with existing electronic health records, (b) simple to extend and update by its users and (c) based on accepted medical vocabularies (ontologies).

In order to maintain a scalable and sharable ontology, the preliminary set of diagnoses (explanations) were recorded as preferred semantic terms from ICD-10 (for clinical diseases) and RxNorm (for medications). Findings (observations) including signs, symptoms, laboratory, and imaging results were gathered from primary data sources and mapped to preferred terms from the SNOMED-CT and LOINC clinical terminologies within the UMLS® Metathesaurus® [32]. In general, demographics and clinical observations were encoded using SNOMED-CT preferred terms whereas laboratory findings were mapped to LOINC.

For a given ICD-10 diagnosis or common medication (A) we described the associated observations (B) as weighted numerical probabilities based upon the frequency of association of B given A.

$$ P\left(A|B\right)=\frac{P\left(B|A\right)P(A)}{P(B)} $$

For instance, if a given disease always presents an associated observation we would weight that with 1.0, if it was associated 10% of the time we would use 0.1, and if that association never occurred, it was encoded with 0. Negating findings, e.g. gender and specific conditions or rash and Malaria were encoded as − 1. When only written descriptions were available we translated them to a numerical value as per Additional file 1. Initial mappings will be refined as public curation is enabled (see Additional file 2). Prior probabilities P(A) were encoded for infectious syndromes and preliminarily assigned binary values based on presence or absence in broad geographic areas. Other binary relations such as sex, required conditions or related diseases were encoded similarly. We encoded age distributions by broad groups; infant (0–6 months), child (6 months-12 years), adult (13–60 years), elderly (> 60).

There are currenly 8221 symptoms in the knowledge base. The most common are ‘fever’(485), ‘headache’(388), ‘nausea’(333) and ‘vomiting’(303).

**Fig. 1:**
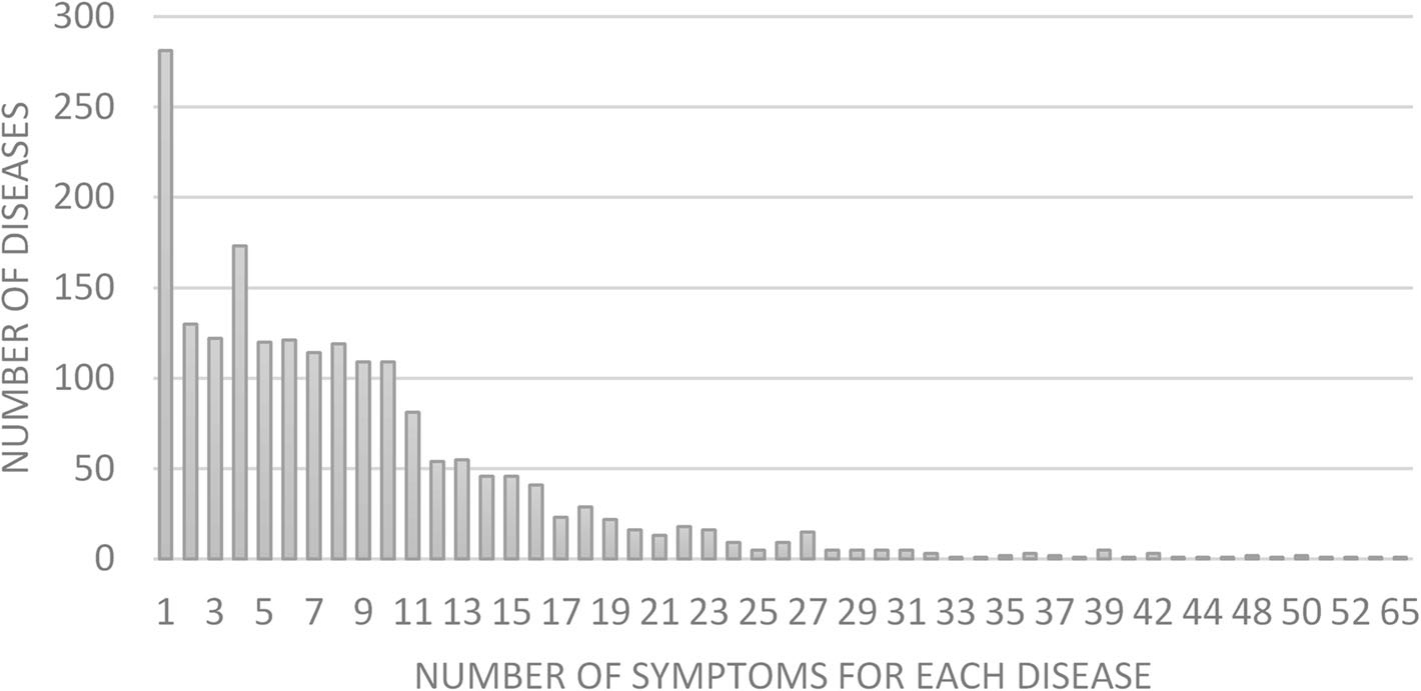
The majority of diseases is described by less then 10 symptoms, but there is a long tail to up to 67 symptoms for single disease

Figure 1 shows that 28% of diseases are described by 10 or more symptoms. The most extensively described diseases are Sarcoidosis (67), Trypanosomiasis (56) and Malaria (55). 42% are defined by 5 or less symptoms and 14% of diseases are described by a single symptom. These single symptom diseases are often self-evident, e.g. contusions or burns. While they don’t hold dignostic challenges, they are included for completeness as they may become part of a differential diagnosis.

## Utility and discussion

The relative quality of the knowledge base was measured by comparing the performance of a simple diagnostic algorithm that draws from this knowledge base. To this end, we developed a first prototype called Doknosis, an interactive differential diagnosis application to parse and visualize the feedback that can be generated using the current state of the KB. We compared DXplain^1^ and Isabel^2^ to Doknosis to evaluate the initial version of the database as these were reported as the “best” performing diagnostic support tools in a recent study [33].

In its current state, the knowledge base provided robust basis for DDSS development and delivered comparable results to established DDSS; performing similar to DXplain and better than Isabel on 117 cases extracted from respected medical journals. The development of the DDSS benefitted from the structure of the database and unearthed several possible improvements such as the inclusion of synonyms and deprecation.

### Doknosis and the set-covering algorithm

**Fig. 2:**
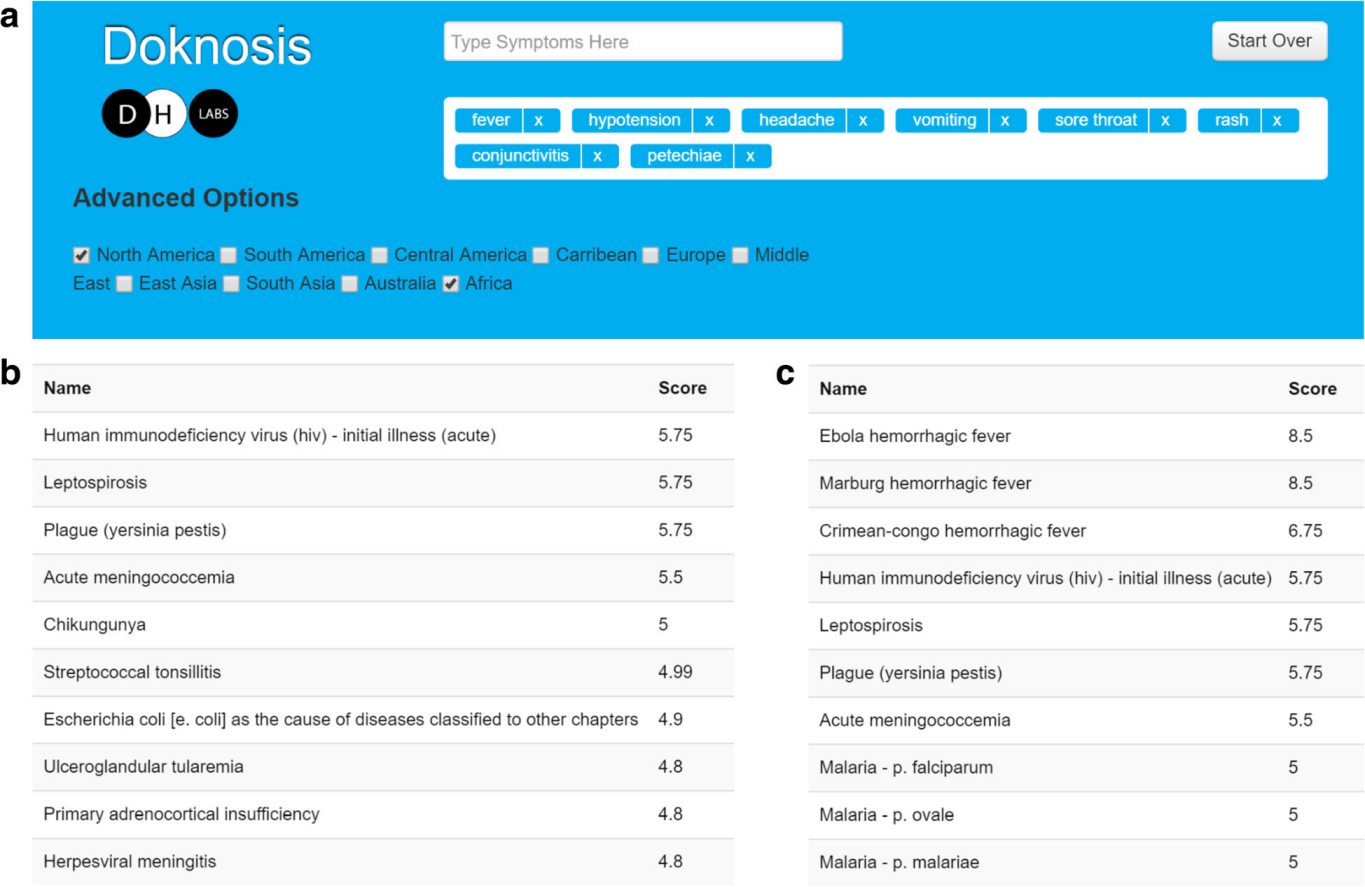
Screenshots of the Doknosis user interface depicting a typical use case and the top 10 explanations for two different options. Up to 20 results can be displayed and are ranked according to their calculated score which grows with the number of related observations. Subfigure (**a**) shows the query interface with the symptoms for an ebola patient, (**b**) shows the resulting list if only North America is selected, and (**c**) depicts the results if Africa is included

Doknosis features a simple user interface to input symptoms using auto-completion, and implements a classic algorithm for finding the best diagnosis for a given set of symptoms. The algorithm is a modified form of set-covering [34] and was used to generate lists of explanations for symptoms extracted from 117 medical case descriptions from trusted journals as shown in Fig. 2.

Formulating the problem of finding the best set of diseases that explain a set of observed symptoms in terms of set-covering was proposed by Reggia et al. [34]. Each disease is associated with a set of symptoms, and the goal is to find the smallest set of diseases for which the union of associated symptoms contains the observed symptoms. In the weighted instance, where each symptom-disease pair gets an association weight, the set-covering objective changes to finding the smallest set of diseases that maximizes the symptom cover weight. Hence, this approach can identify multiple overlapping explanations that contribute to the set of symptoms. A diagnosis can be either a single medical disease, a medication side effect, or can contain multiple disease explanations, e.g. HIV and Pneumocystis Pneumonia or Influenza and side effect of Tamiflu.

The associations between symptoms and diseases in the database we collected were given a weight *w*(*s*,*d*)∈[0,1] that for each symptom - disease pair (*s*,*d*) reflected their recorded association fraction (percent/100%). A greedy algorithm is applied where at each step the disease *d* is chosen that maximizes *m*(*D*∪{*d*}) − *m*(*D*).

### Dataset extraction

For validation and comparison, a set of 117 unique medical cases was extracted from published case reports and medical study aids. Initially, 150 case reports were selected from trusted journals such as the New England Journal of Medicine (NEJM), The American Journal of Tropical Medicine (AJTM), and from UWorld question bank (www.uworld.com) used by students studying for the United States Medical Licensing Examination (USMLE). While medical case reports often represent rare or interesting presentations of diseases, medical question banks are written by board-certified experts and are typically peer-reviewed for accuracy. We also collected various cases (OTHER) from further journals. A full overview of the cases and journals can be found in Additional file 3. A subset of cases were chosen with particular emphasis on febrile and infectious syndromes given our platform’s history as a diagnostic aid in Mozambique. Other basic categories were meant to address the most common presenting syndromes reported in the medical literature. A list of all used search terms can be found in Additional file 4.

Three datasets of 50 cases each were created by randomly sampling from these sources. *Dataset1* contains cases from NEJM, *Dataset2* from UWorld and *Dataset3* was formed from AJTM and OTHER, here with a bias toward febrile syndromes. For each case within the three datasets, two evaluators reviewed the medical cases, reports and/or journals to assess the quality of the case and extract a concise list of demographics, signs, symptoms and tests. For the purposes of this work, medications and multiple disease explanations were excluded. While performing this task, evaluators were not allowed to discuss with each other. Evaluators indicated whether each case was considered *rare* or *common* and also indicated whether determining the correct diagnosis for a trained medical expert under ideal circumstances was *very difficult* or *average*. Thirty-three cases were flagged for exclusion if there was an unknown diagnosis, or the diagnosis was not found in ICD-10. Our evaluation considered only those 117 cases which none of the evaluators had excluded.

Evaluators rated the New England Journal of Medicine cases as most difficult and categorized 50% of them as rare cases. The UWorld and AJTM dataset were rated comparably with 30% rare cases and a slightly higher difficulty in the UWorld cases. The three datasets and the estimated difficulty and prevalence of contained cases are summarized in Table 1.

**Table 1:** Prevalence and difficulty of the cases selected

Dataset	#cases	%Very Difficult	% Rare
1(NEJM)	24	42%	50%
2(UWorld)	43	33%	30%
3(AJTM+OTHER)	50	26%	30%

### Evaluation procedure and data analysis

Six medical practitioners entered the abstracted signs or symptoms into Doknosis, DXplain and Isabel with the enforcement of auto-complete from the given platform. Evaluators made notes where auto-complete failed to match an input term or a clear synonym was unavailable. The rank of the correct diagnosis was then recorded for each of the cases.

To compare the Doknosis results with the results obtained with Isabel and DXplain, we grouped the reported results into Top 1, Top 10 and Top 20. For example, if an evaluator reported a rank of 3 for a case this would fall under bucket Top 10. Top n represents the number of cases in which the right diagnosis was present in the top n results returned by the tool. The ranking can be impacted if several diseases have the same score. Hence, a disease may by cut-off from the “top-n” despite having the same likelihood scores as other diseases in the “top-n”. An overview and the detailed ranking results can be found in Additional file 5 and Additional file 3. The utility function used here maps the ranking of the correct explanation to a score value between 0 and 3. If the correct explanation is shown first (Top1) the score is 3. Responses in the Top10 result in a score of 2 and Top20 in a score of 1. The score is 0 if the answer is not shown in the Top20. Score differences were analyzed using Wilcoxon signed-rank test.

### Performance comparison

Doknosis and DXplain performed comparibly but both provided significantly better results than Isabel (Z = 2.44, *p* < 0.014). DXplain outperformed Doknosis on the NEJM dataset but Doknosis excelled in the tropical diseases. Overall Doknosis performed insignficantly (7%, *p* = 0.49) better than DXplain. Table 2 shows the differences in ranking results and the resulting score for the three tools for each dataset and across all datasets. More detailed results can be found in Additional file 5 and Additional file 3.

**Table 2:** Comparison of Doknosis in set-covering mode vs. Isabel and DXplain analyzing test cases from the three different datasets

	NEJM (24)			UWorld (43)			AJTM + OTHERS (50)			Overall (117)		
	Top 1	Top 20	Score	Top 1	Top 20	Score	Top 1	Top 20	Score	Top 1	Top 20	Score
Doknosis	8%	50%	1.00	28%	42%	1.07	50%	92%	2.30	33%	65%	1.57
Isabel	4%	50%	0.96	14%	37%	0.86	22%	70%	1.58	15%	54%	1.19
DXplain	21%	58%	1.21	23%	47%	1.09	48%	76%	1.90	33%	62%	1.46

Despite the use of a rather simple set-covering algorithm, the Doknosis prototype performed comparably to the accuracy of the established programs in each category (top 1, 20). These results could be partly due to a bias towards diseases from a specific domain but are surprising given the differences in sophistication. Doknosis excelled in the category of infectious diseases and tropical medicine (Dataset3) and showed the quality of the database and simple parsing algorithm is comparable to existing tools in the current core topics. However, there is significant room for improvement in both finding the best single explanation and likewise presenting the best differential diagnosis.

### Platform quality

Doknosis was not developed as a decision support system but as means to develop new algorithms and evaluate the quality and completion, build up and curate the knowledge base. Nevertheless, the development and evaluation of Doknosis provides insights into the qualities of the current knowledge base as a platform for future DDSS prototypes.

A lack of support for synonyms was a major hurdle to usage of the system. For instance, Doknosis did not understand *Shortness of Breath* or *SOB* but expected *dyspnea* in some cases whereas either could be entered in other cases. This is a direct consequence of the current structure of the knowledge base and led to work including synonyms as well as curation to insure use of preferred terms. We expect more challenges to surface as more prototypes build on the knowledge base.

While the current database contains more than 2000 unique explanations, there remained missing diseases and findings discovered during initial testing. Given the tool’s history, these tended to be rare conditions most often encountered in the developed world, with better coverage of infectious and emerging tropical diseases. Continued validation and updating of relations must be carried out before the tool can be considered for prospective clinical testing or public use. The current knowledge base can be extended through crowd-sourcing and complemented with data generated by machine learning, both approaches that are underway (see Additional file 2). Likewise, knowledge-based algorithms could accompany machine learning approaches either as a source of ground truth or as a topical layer that could be used to foster interaction or improve explainability.

### Limitations

This paper is primarily meant to demonstrate the feasibility of mapping associations with preferred terms in a UMLS based ontology, to act as an open platform for prototyping DDSS. The knowledge base is still incomplete, does not support synonyms, is yet to fully account for multiple concurrent diseases, and the handling of negative findings is rudimentary. The current format does not account for severity of presentation, and cannot represent typical presentation trajectories. Likewise our set-covering algorithm, while it does make use of edge weights has significant drawbacks such as performance, and the inability to require key findings. Ultimately we hope the knowledge base will grow and take full advantage of the UMLS structure (and related ontologies) by utilizing mappings such as synonyms, deprecation and related terms.

## Conclusion

In this article, we discuss the construction and preliminary testing of an open access medical knowledge base intended to spur the development of digital medical cognitive assistants. Our first prototype performed comparably to commercial applications, however in-depth testing revealed both missing diseases and symptoms, as well as issues with synonym utilization and redundancy. These topics are being addressed in revisions to the knowledge base.

For the near future, we propose medical experts working with technology (human technology teams) will remain superior to any purely technical intervention. Technology can assist cognitive activities that are naturally difficult like Bayesian reasoning, make providers or patients better thinkers, or aid in the analysis of complex data. Moreover, knowledge-based systems may be needed to collaborate, explain and mediate between machine learning algorithms and human users.

## Additional files

Additional file 1: Initial Mapping of Likelihood Scores for Symptoms and Signs. (PDF 13 kb)

Additional file 2: Administration interface of the knowledge base. (PNG 151 kb)

Additional file 3: Evaluation datasets and results. (PDF 53 kb)

Additional file 4: Search terms for case retrieval. (PDF 13 kb)

Additional file 5: Overview of evaluation results. (PDF 79 kb)

## Abbreviations

AI: Artificial Intelligence
AJTM: American Journal of Tropical Medicine
DDSS: Diagnostic Decision Support System
KB: Knowledge Base
ML: Machine Learning
NEJM: New England Journal of Medicine
UMLS: Unified Medical Language System

## Footnotes

1
http://www.DXplain.org/

2
http://symptomchecker.isabelhealthcare.com/
