# Supplementary material for: An open access medical knowledge base for community driven diagnostic decision support system development
Source: BMC Med Inform Decis Mak. 2019 Apr 27;19:93. doi: 10.1186/s12911-019-0804-1 (PMC6486985; doi:10.1186/s12911-019-0804-1)
Supplement: Supplementary file 1 — Initial Mapping of Likelihood Scores for Symptoms and Signs. (PDF 13 kb) [file 12911_2019_804_MOESM1_ESM.pdf]

Appendix A: Initial Mapping of Likelihood Scores for Symptoms and Signs

CODE	DESCRIPTORS	PERCENTAGE
1.0	Always, all	100%
0.75	Commonly, often	75%
0.5	Probable	50%
0.25	Infrequently, uncommonly	25%
0.1	Rarely	10%
0.0	Never	0%

Table 1: Initial mapping of likelihood scores for symptoms and signs
