# Supplementary figures and images for: An open access medical knowledge base for community driven diagnostic decision support system development
Source: BMC Med Inform Decis Mak. 2019 Apr 27;19:93. doi: 10.1186/s12911-019-0804-1 (PMC6486985; doi:10.1186/s12911-019-0804-1)

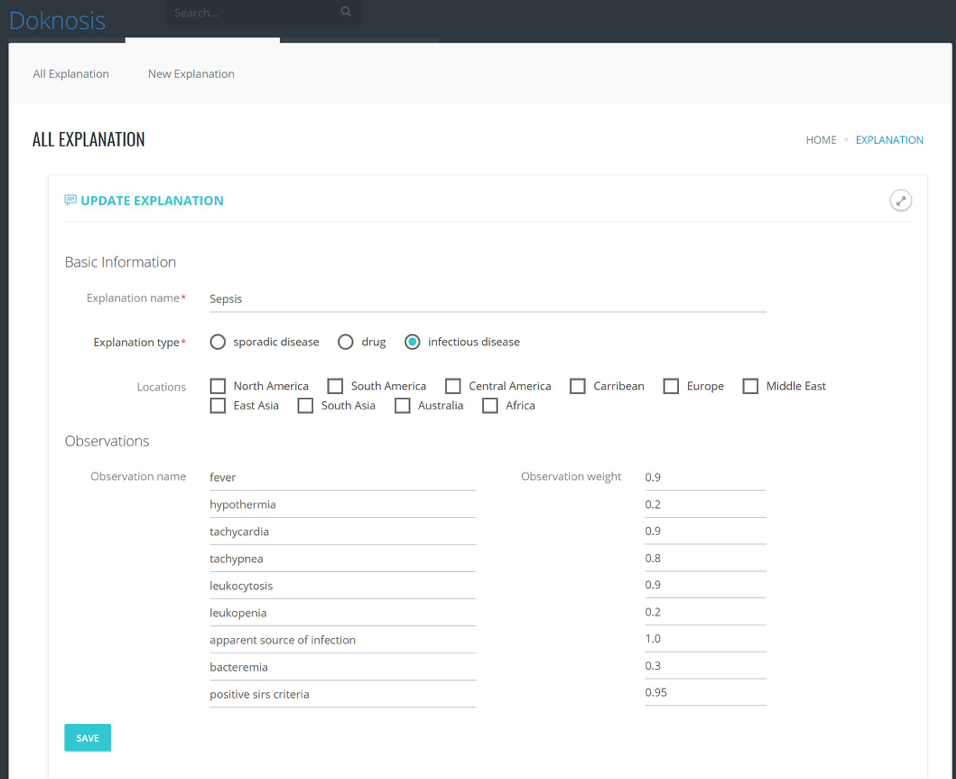

Supplement: Supplementary file 2 — Administration interface of the knowledge base. (PNG 151 kb) [file 12911_2019_804_MOESM2_ESM.png]
