# Supplementary material for: An open access medical knowledge base for community driven diagnostic decision support system development
Source: BMC Med Inform Decis Mak. 2019 Apr 27;19:93. doi: 10.1186/s12911-019-0804-1 (PMC6486985; doi:10.1186/s12911-019-0804-1)
Supplement: Supplementary file 3 — Evaluation datasets and results. (PDF 53 kb) [file 12911_2019_804_MOESM3_ESM.pdf]

Appendix D: Evaluation Datasets and Results

DOI	*Extracted Symptoms*	*Answer*	*Rare*	*Very Difficult*	*Doknosis*	*ISABEL*	*DXplain*
10.1056/NEJMcpc1305994	Fever, abd pain (LLQ), lymphoma, fatigue, weight loss, immunosuppression, thrombocytopenia	Neutropenic enterocolitis and appendicitis	no	no	1	>20	>20
10.1056/NEJMcpc1305994	Abd pain, bloating, rectal bleeding, weight loss, anxiety, disruptive thoughts, suicidality	GAD	no	no	>20	>20	4
10.1056/NEJMcpc1305990	Night sweats, abd pain (pleuritic), nausea, loose stools, lymphadenopathy (inguinal), plaques, leukopenia, neutrophilia, (elevated ACE)	Sarcoidosis / Lymphoma	no	no	3	1	1
10.1056/NEJMcpc1411928.	Cough, nasal congestion, post-tussive emesis, post-tussive SOB, epistaxis	Pertussis	no	no	3	16	1
10.1056/NEJMcpc1200090	Chest pain, (radiation to neck,) dyslipidemia, lung crackles, bradycardia, st elevation	MI	no	no	2	2	1
10.1056/NEJMcpc1407131	Fever, chest pain, chills, urticaria, chest pain, (llsb murmur,) inguinal lymphadenopathy, ivdu, tachycardia, lbp (HCV infection and substance use disorder)	Endocarditis	no	no	4	4	4
10.1056/NEJMcpc1007084	Fever, abdominal pain, nausea, foul smelling urine	EBV	no	no	>20	13	>20
10.1056/NEJMcpc1400841	Dyspnea, hypoxia, tachypnea, lymphocytopenia	PCP PNA, HIV	no	no	2	4	19
10.1056/NEJMcpc1304165	Fever, upper abd pain, weight loss, nausea, vomiting, tachycardia, firm abdomen, neck lymphadenopathy, lung nodules	HIV, (miliary TB), disseminated histoplasmosis	no	yes	5	>20	>20

10.1056/NEJMcpc1400842	Abd cramping, hematochezia, bloody diarrhea, back spasms, joint pain, fevers, weakness, weight loss, night sweats	UC, CMV infective colitis	no	yes	13	3	1
10.1056/NEJMcpc1410936	Abd pain, dyspnea, diplopia, dysphagia, proximal extremity weakness	Botulism	yes	no	5	>20	2
10.1056/NEJMcpc1404517	Weight loss, back pain & tenderness, foot numbness, iritis, ankle pain, knee swelling	Ankylosing Spondylitis	yes	no	>20	6	11
10.1056/NEJMcpc1404140	Fever, rash, pancytopenia, abnormal liver tests, fatigue, headache, sore throat, arthralgias, sore throat, truncal pigmented macules, cervical lymphadenopathy	Measles (would need to specify age)	yes	no	>20	10	>20
10.1056/NEJMcpc0902223	Fever, night sweats, diffuse pruritic maculopapular rash, neck & axillary lymphadenopathy, splenomegaly	T-cell Lymphoma	yes	no	>20	5	3
10.1056/NEJMcpc1410939	Abd pain, syncope, hypotension, flushing, blurry vision, perioral & hand tingling, diaphoresis, urinary incontinence, hypotension, AMS, nausea, hand swelling	Anaphylaxis from hydatid cyst	yes	yes	>20	>20	16
10.1056/NEJMcpc1400835	(Left) chest pain, (decreased lung sounds,) Calf swelling, pleural effusion	Pleural malignant tumor (mesothelioma or metastatic lung adenocarcinoma)	yes	yes	>20	>20	14
10.1056/NEJMcpc1400838	Headache, fever, stiff neck, AMS, nausea, syncope, drowsy, truncal ataxia	(migraine), mycoplasma pneumonia meningitis	yes	yes	20	4	1
10.1056/NEJMcpc1210260	Fever, myalgias, arthritis, erythematous rash, heroin use, joint pain, cough, malaise, joint swelling, weakness	serum sickness from acute Hep B	yes	yes	>20	>20	4
10.1056/NEJMcpc1410935	Sore throat, fatigue, hoarseness, facial edema, facial swelling, periorbital edema, fatigue	Hypothyroidism, statin myopathy	yes	yes	>20	>20	>20
10.1056/NEJMcpc1305985	Africa, dyspnea, cough, raised violaceous lesion, HIV, malaise, fatigue, weight loss, tachycardia	Kaposi's sarcoma, TB, immune reconstitution inflammatory syndrome	yes	yes	3	>20	>20

10.1056/ NEJMcpc 1305992	SOB, fever, CP, cough, DOE, early satiety, hoarseness, leg swelling, dysphagia, tachycardia	PAH	yes	yes	>20	>20	>20
10.1056/ NEJMcpc 1209304	Child, fever, abdominal pain, dysuria, oliguria, lethargy, diarrhea, pelvic mass	Burkitt's	yes	no	>20	>20	>20
10.1056/ NEJMcpc 1400841	SOB, DOE, hypoxia, tachypnea	HIV, pneumocystis pneumonia, interstitial lung disease	no	no	1	2	>20
10.1056/ NEJMcpc 1415165	Fever, headache, petechial rash, nausea, diffuse myalgias, neck stiffness, nuchal rigidity, (elevated) d-dimer,	Neisseria meningitis with complement deficiency	no	yes	>20	>20	>20

Table 1: Dataset 1 - New England Journal of Medicine

Case ID	*Extracted Symptoms*	*Answer*	*Rare*	*Very Difficult*	*Doknosis*	*ISABEL*	*DXplain*
2868	lethargy, easy fatigability, pallor, splenomegaly, palpable spleen, palpable splenomegaly, atypical lymphocytes, leukocytosis	hairy cell leukemia	yes	no	4	1	>20
3083	constipation, abdominal pain, (obese) obesity, weight loss, history of atrial fibrillation, dehydration, multiple daily vitamins, (warfarin, diltiazem, numerous daily vitamins and minerals)	vitamin d overdose (=hypervitaminos is D)	yes	yes	1	>20	>20
10763	dyspnea, cough, dry cough, smoking history, alcohol, alcoholism, alcohol use, history of alcohol abuse, history of heavy alcohol use, history of chronic alcohol consumption, elevated creatinine, serum creatinine raised, seizure, hypertension, hypertensive crisis, severe hypertension, confusion, agitation, altered mental status	nitroprusside (cyanide) toxicity	yes	yes	14	>20	>20
2713	weakness, hemiparesis, fatigue, fever, low-grade fever, low fever, low grade fever, palpitations, weight loss, murmur, diastolic murmur, (left atrial mass)	intracardiac tumor (cardiac myxoma)	yes	yes	>20	>20	>20

2233	adolescent, hematuria, lower abdominal pain, pelvic or lower abdominal pain, abdominal pain, deafness in child, deafness, proteinuria, protein in ua, renal failure, child	alport syndrome	yes	yes	>20	>20	>20
3582	diarrhea, abdominal pain, weight loss, bulky diarrhea, foul-smelling diarrhea, abdominal distension, abdominal distention, flatulence, arthralgia, polyarthralgia, chronic cough, cough, lymphadenopathy, generalized lymphadenopathy, hyperpigmentation, hyperpigmentation of skin, diastolic murmur, pas-positive macrophages in intestinal biopsy	whipple's disease	yes	yes	1	5	1
4388	fever, chills, pleuritic chest pain, dyspnea, positive hiv pcr, positive hiv elisa, positive hiv western blot, tachycardia, crackles on lung exam, crackles in adult, diffuse crackles, crackles, leukocytosis, lung cavitations	s aureus endocarditis	no	yes	>20	>20	>20
4436	african american, nocturia, recurrent otitis media, sexually active, family history of the disease	family history (sickle cell trait)	no	yes	>20	>20	>20
3940	adolescent, child or adolescent, adolescent or young adult, facial swelling, fatigue, loss of appetite, decreased appetite, decrease in appetite, "history of travel to southeast asia and australia, or middle east, india or china", "history of travel to china, india, malaysia, the philippines, indonesia, or various pacific islands", china, travel to china, periorbital edema, hypoalbuminemia, decreased albumin, decreased serum albumin, hepatitis b infection, proteinuria, nephrotic proteinuria	membranous nephropathy	no	yes	>20	2	1
3061	white cell casts in urine, arthralgia, rash, recent antibiotic use, fever, sexually active, dysuria, polyuria, urinary frequency, (increased urinary frequency,) acute renal failure, proteinuria, mild proteinuria, maculopapular rash	Drug-induced interstitial nephritis	no	yes	17	5	7
10670	bump, nonpruritic skin lesions, palpable nodule, subcutaneous nodules, nontender firm subcutaneous nodules	epidermal inclusion cyst	no	yes	>20	>20	>20

4497	infant, ventricular septal defect, stridor, murmur, holosystolic murmur loudest at left sternal border, systolic murmur, pansystolic murmur, (stridor improves with neck extension)	vascular ring	yes	yes	>20	>20	>20
4239	child, acne, (advanced bone age, coarse axillary and pubic hair, severe cystic acne, low LH, negative GNRH stimulation, tall)	nonclassic congenital adrenal hyperplasia (21-hydroxylase deficiency, gonadotropin independent (peripheral))	yes	yes	>20	>20	>20
2664	slurred speech, trouble walking, difficulty walking, drowsiness, (history of) insomnia, (history of) migraine, (history of) migraines, history of epilepsy, lethargy, hyporeflexia, (bipolar disorder, history of hypothyroidism, reactive pupils)	benzodiazepine overdose	no	yes	>20	>20	>20
3396	infant, seizure, grand mal seizure, aka tonic-clonic seizure, seizure in infant, full-term, retinal hemorrhage, (increased head circumference)	shearing of subdural veins (non-accidental trauma)	yes	yes	>20	>20	>20
3769	pulmonary fibrosis,pneumonitis	amiodarone (toxicity)	no	no	>20	>20	>20
3872	newborn, difficulty feeding, poor feeding, cyanosis, dyspnea	Choanal atresia	yes	no	>20	>20	>20
4114	nodules on cxr, nodules on chest x-ray, lung nodules, (mississippi, southern united states, nonsmoker)	histoplasmosis	no	no	>20	1	4
3146	solitary, poor eye contact, (not bothered)	schizoid personality disorder	no	no	1	>20	>20
2746	rash, rash on trunk, itching, itchiness, itchy rash, sexually active	tinea corporis	no	no	>20	>20	>20

4635	sexual dysfunction, male, impotence, weight loss, gynecomastia, low t3, low t4, (chronic) hypogonadism, testicular atrophy	chronic liver disease (alcoholic cirrhosis)	no	no	>20	>20	>20
3379	child, male, behavioral changes, poor eye contact, restricted behaviors, solitary	Autism spectrum disorder	no	no	>20	>20	>20
3951	dyspnea on exertion, palpitations, widened pulse pressure	aortic regurgitation	no	no	>20	>20	1
3574	shoulder pain, history of anterior shoulder pain exacerbated by lifting or elevated pushing or pulling of objects	subacromial bursitis	no	no	>20	>20	>20
4253	pain behind the eyes, eye pain, severe eye pain, sharp or stabbing pain, alcoholism, ptosis, ptosis with miosis (partial horner syndrome), miosis, pain awaking patient at night, sudden awakening, intense pain, retroorbital pain	cluster headache	no	no	1	1	1
4434	abdominal pain, epigastric pain, acute onset of epigastric pain, vomiting, improved by sitting up and leaning forward, hyperlipidemia, smoking history, alcohol use, alcohol, alcoholism, left sided pleural effusion	acute pancreatitis	no	no	1	1	1
2860	eye pain, red eye, eye redness, watery eyes, watering of the eye, vesicles, dendritic ulcer, corneal ulceration	herpes simplex keratitis	no	no	1	3	4
4897	paranoia, delusion, nonbizarre delusions	delusional disorder	no	no	1	6	2
4772	loss of consciousness, trouble walking, difficulty walking, skin infections, diabetic, diabetes mellitus, hypotension, shock, tachycardia, (cold, clammy skin), cool clammy skin, pulmonary hypertension, syncope, (normal pcwp)	pulmonary embolism	no	no	>20	7	1
4306	weight loss, fever, cough, sputum production, productive cough, nausea, abdominal pain, dizziness, (postural dizziness, subnormal cortisol rise with cosyntopin stimulation test, bilateral adrenal calcification)	tuberculosis	no	no	>20	15	17

2452	bilious vomiting, newborn, vomiting, maternal polyhydramnios, delayed passage of meconium, hypotonia, enlarged tongue, (protruding tongue), up-slanting palpebral fissure, low-set ears, fifth finger clinodactyly, holosystolic murmur loudest at left sternal border, thrill, precordial thrill, down syndrome	duodenal atresia	no	no	1	>20	2
4486	central america, travel to central america, nausea, vomiting, suicidality, suicidal behavior, diabetes mellitus - type 2, diabetes mellitus, smoking history, tachycardia, dehydration, (presence of a gastric splash, hear four hours or more after the last meal,) peptic ulcer disease, vomiting of partially digested food, history of acid ingestion, early satiety	pyloric stricture	no	no	1	>20	1
4463	anorexia, nausea, weakness, amiodarone, atrial fibrillation, history of atrial fibrillation, furosemide, digoxin	drug interaction (digoxin)	no	no	0	>20	>20
4331	african american, "african american (in us)", fever, jaundice, abdominal pain, dark urine, tachycardia, (bite cells on peripheral smear, RBC inclusions on crystal violet stain)	G6PD Deficiency	no	no	>20	>20	>20
3959	slurred speech, hand weakness, focal weakness, history of myocardial infarction, aspirin use, other known associations include penicillins, cephalosporins, enalapril, rifampin, nsaids, smoking history, hypertension, dysarthria	lacunar stroke (hypertension)	no	no	>20	>20	15
2901	liver disease, fatigue, ascites, furosemide, (history of) esophageal varices, beta blocker, (beta-blocker use,) tachycardia, abdominal pain, ascites, elevated cell count in ascitic fluid (abdominal discomfort, history of chronic ascites, history of alcoholic liver disease, ascitic fluid with high neutrophils, high serum-ascites albumin gradient (SAAG), no free air on upright cxr)	spontaneous bacterial peritonitis	no	no	1	1	1
4118	fever, productive cough, putrid sputum, recurrent pneumonia, smoking history, alcoholism, history of alcohol abuse, history of heavy alcohol use, infiltrates on cxr, hypoxemia, consolidation on cxr, crackles, crackles in adult, crackles on lung exam, dyspnea	aspiration pneumonia	no	no	1	3	13
3037	midwestern united states, fever, night sweats, cough, productive cough, weight loss, subacute, subacute onset, rash, skin lesion, skin lesions, verrucous skin lesions, lytic lesions on bone films, osteolytic lesions, consolidation on cxr	blastomycosis	no	no	2	>20	>20

4698	upper extremity atrophy, trauma, history of trauma, weakness	Syringomyelia	yes	no	1	7	>20
3815	oral ulcers, blurry vision, vision changes, anterior uveitis, uveitis, genital ulcers, multiple painful genital ulcers, nodular lesions, hypopigmented lesions	behcet's disease	yes	no	>20	>20	1
4241	presentation with isolated knee or thigh pain, pain with walking, pain on walking, history of copd, smoking history, visible bulge in the groin, (pulsatile groin mass, anterior thigh pain)	femoral artery aneurysm	yes	no	>20	>20	>20
3548	child, polydipsia, polyuria, incontinence, urinary incontinence, incontinence of bladder, fatigue, dehydration, (enuresis)	autoimmune destruction of the pancreatic beta cells (DMI)	no	no	4	1	2
3679	vulvar burning, vulvar pruritis, vaginal discharge, green vaginal discharge, frothy vaginal discharge, thin vaginal discharge, (ph 5.5)	flagellated motile organisms	no	no	2	2	1

Table 2: Dataset 2 - UWORLD

Source	*Extracted Symptoms*	*Answer*	*Rare*	*Very Difficult*	*Doknosis*	*ISABEL*	*DXplain*
Am J Trop Med Hyg. 1992 Feb;46(2):146-50.	patient younger than 45 years, african american, farmer, south america, weakness, cough, fever, weight loss, myalgia, dyspnea, shaking chills, bilateral diffuse pulmonary infiltrates on cxr, honeycombing on cxr, granulomas on chest x-ray, fungus present on microscopy	Fulminant disseminated pulmonary adiaspiromycosis	yes	yes	>20	>20	>20
Am J Trop Med Hyg. 1992 Feb;46(2):161-4.	female, adolescent or young adult, travel to north africa, fever. diarrhea, chills, peripheral neuropathy. myalgia, exposure to ticks, tick bite, arthralgia, elevated erythrocyte sedimentation rate (esr) (esr)	Ehrlichiosis	no	no	7	>20	2

Am J Trop Med Hyg. 1992 Jul;47(1):10-2.	young adult, travel to southeast asia, history of travel to china, india, malaysia, the philippines, indonesia, or various pacific islands, male, dyspepsia, abdominal pain, diarrhea, headache, fatigue, weight loss, fever, pruritus, ingestion of raw fish, abdominal tenderness, tender hepatomegaly, (eosinophiliaxova present in stool)	Acute amoebic dysentery (capillariasis--capillaria philippinesis)	no	no	5	>20	>20
Am J Trop Med Hyg. 1991 Dec;45(6):723-7.	male adult human immunodeficiency virus (hiv) positive t. Cruzi, fever, headache, brazil, hemophilia, hepatomegaly, splenomegaly ,leukopenia,	Chagas' disease (trypanosoma cruzi)	no	no	1	17	>20
Am J Trop Med Hyg. 1989 Mar;40(3):323-5.	africa, infant, fever, cough, chronic diarrhea, lymphadenopathy ,gingivitis, stomatitis, hepatomegaly, splenomegaly, hepatosplenomegaly, painful violaceous papule, skin nodules,positive hiv elisa	Acquired immune deficiency syndrome (aids)--Lympho-cutaneous Kaposi's sarcoma	no	yes	11	10	1
Am J Trop Med Hyg. 1981 Jan;30(1):145-8.	brazil, travel to south america, female, young adult, fever, chills, headache, abdominal pain, arthralgia, myalgia, jaundice, epigastric tenderness, hyperbilirubinemia, elevated ast and alt, uremia, elevated erythrocyte sedimentation rate (esr), albuminuria, sterile pyuria, negative leptospirosis serology, negative cmv serology, flavivirus	St louis encephalitis	yes	yes	>20	>20	>20
Am J Trop Med Hyg. 1984 Nov;33(6):1155-8.	adult, male, history of travel to china, india, malaysia, the philippines, indonesia, or various pacific islands, fresh water exposure, recent skin contact with fresh water, fever, headache, nausea, vomiting, visual changes, hepatomegaly ,eosinophila ,leukocytosis, gradually increasing headache and confusion, elevated erythrocyte sedimentation rate (esr), elevated lactate dehydrogenase (ldh), elevated ast and alt, csf eosinophilia, schistosomiasis ova in stool	Schistosomiasis - mansoni (japonica-in case report) (acute)	no	no	8	10	>20
Am J Trop Med Hyg. 1982 Mar;31(2):226-9.	male, adolescent or young adult, contact with cattle, contact with farm animals, fever, malaise, nausea, abdominal pain, anorexia, diarrhea, positive cryptosporidium naat of stool	Cryptosporidiosis	no	no	8	>20	1

Am J Trop Med Hyg. 1996 Dec;55(6):584-5.	adult history of travel to china, india, malaysia, the philippines, indonesia, or various pacific islands diarrhea weight loss female malaise nausea anorexia fatigue fungal infection elevated erythrocyte sedimentation rate (esr) (esr) cyclospora in stool smear	Cyclosporiasis (cyclospora infection)	no	no	1	>20	2
Am J Trop Med Hyg. 1999 Jul;61(1):34-6.	asian descent, travel to asia, travel to southeast asia, southeast asia, male adult, fever, abdominal pain, left upper quadrant abdominal pain, walking barefoot, chills, history of diabetes, hyperglycemia, elevated ast, elevated alkaline phosphatase, interstitial infiltrates on cxr, adrenal gland involvement, adrenal enlargement, burma, burkholderia pseudomallei	Melioidosis	yes	yes	1	>20	>20
Am J Trop Med Hyg. 2006 May;74(5):901-4.	taiwan, recent travel to southeast asia or latin america, travel to southeast asia, acute abdomen, thrombocytopenia, lymphocytosis, elevated aptt, elevated pt, elevated alt and ast, fever, myalgia, headache, petechial rash	Dengue shock syndrome (dengue hemorrhagic fever)	no	yes	7	4	2
Am J Trop Med Hyg. 2006 Aug;75(2):303-6.	maleage over 40, fever, fatigue, headache, contact with farm animals, farmer, neurologic symptoms, leukocytosis, eosinophilia, lung nodules, elevated ige, increased ige, ingestion of raw poultry, positive toxocara serology, ascaris larvae in tissuecough	Toxocariasis (visceral larva migrans)	no	no	1	>20	1
Am J Trop Med Hyg. 2007 May;76(5):886-7.	young adult, young adult and adolescent, adolescent or young adult, male, south america, fever, abdominal pain dyspnea, hypotension, tachycardia, tachypnea, hypoxia. bilateral diffuse infiltrates on cxr, hypoxemia, hyperglycemia, elevated serum lipase, elevated serum amylase, elevated serum creatinine, elevated alt and ast, hyperbilirubinemia, conjugated hyperbilirubinemia, elevated alkaline phosphatase, increased creatinine phosphokinase, leukocytosis, thrombocytopenia, hepatomegaly, positive leptospira microscopic agglutination test (mat)	Leptospirosis	yes	yes	2	>20	>20

Am J Trop Med Hyg. 2007 Nov;77(5):891-2.	maleage 30-60, thailand, tick bite, fever, myalgia, conjunctival injection, petechial rash, weakness, generalized edema, thrombocytopenia, elevated alt and ast, elevated alkaline phosphatase, positive rickettsia serology	Spotted fevers - rickettsial	yes	no	2	2	1
Am J Trop Med Hyg. 2007 Dec;77(6):1096-8.	male, adult, aids, history of aids, headache, blurred vision, fever, ring enhancing lesion on imaging	Brain abscess (disseminated balamuthia mandrillaris amoeba infection)	no	no	1	4	1
Am J Trop Med Hyg. 2008 Jan;78(1):7.	female, india, history of travel to china, india, malaysia, the philippines, indonesia, or various pacific islands, fever, myalgia, headache, melena, hepatomegaly, abdominal distension, thrombocytopenia. positive dengue serology	Dengue shock syndrome	no	yes	1	1	1
Am J Trop Med Hyg. 2008 Jan;78(1):8>200.	male age greater than 50, fever chills, bone pain, gum bleeding, thrombocytopenia, travel to asia, stocking and glove distribution of sensory neuropathy, nausea, diarrhea, cough, bloody sputum, maculopapular rash, petechial rash, testicular swelling, elevated ast and alt, hematuria, positive dengue serology, positive dengue elisa	Dengue shock syndrome	no	yes	9	6	1
Eur J Intern Med. 2008 Mar;19(2):	adult, male, diarrhea, fever, pericarditis, bloody diarrhea, travel to africa. abdominal pain, elevated lactate dehydrogenase (ldh), positive shigella stool culture	Shigellosis	no	no	6	2	15
Indian J Med Microbiol. 2009 Jan-Mar;27(1):65-6.	male, india, history of travel to southeast asia and australia, or middle east, india, or china, fever, adult, chills, diarrhea, altered mental status, tachypnea, weakness, hepatomegaly ,elevated erythrocyte sedimentation rate (esr) (esr), pyuria, resistance to malaria, positive salmonella pcr, bloody diarrhea	Typhoid and enteric fever (salmonella enterica serotype isangi)	no	no	1	4	>20
Am J Trop Med Hyg. 2010 Mar;82(3):371-5. doi: 10.4269/ajtmh.	male, adult, endemic to sub-saharan african or south america, africa, farmer, fever, arthralgia, nausea, vomiting, scleral icterus, abdominal pain, right upper quadrant abdominal pain, hematemesis, malaise	Rift valley fever	yes	no	>20	>20	>20

Am J Trop Med Hyg. 2010 Jul;83(1):10 2-3. doi:	age younger than 60, africa, dyspnea, hacking cough, headache, facial edema, cardiac disease, superior vena cava syndrome, positive echinococcus serology	Echinococcosis - pulmonary cysts (cardiac hydatidosis)	no	no	10	>20	>20
Am J Trop Med Hyg. 2011 Jun;84(6):84 3-4. doi:	young adult, male, fever, chills, dry cough, cough, flank pain, oliguria, elevated creatinine, sterile pyuria, lung nodules, lung cavitations, lymphadenopathy, south america, positive tuberculosis elispot	Tuberculosis	no	yes	2	5	1
Am J Trop Med Hyg. 2011 Nov;85(5):952-6. doi: 10.4269/ajtmh.2011.11-0165.	maleage greater than 60,australia, southeast asia, fever, elevated c-reactive protein, neutrophilia, altered mental status, myoclonic jerks, hypertonia, cogwheel rigidit elevated protein concentration of csf csf pleocytosis xc sf lymphocytosis history of hypertension	West nile fever - neuroinvasive	yes	yes	1	>20	12
Am J Trop Med Hyg. 2012 Jan;86(1):	male adult rice water diarrhea fever fresh water exposure	Cholera	no	no	3	>20	6
Chang Gung Med J. 2011;34(6 Suppl):52-5.	male, elderly, mediterranean and central european location, europe, fever, chills, diaphoresis, malaise, anorexia, weight loss, nausea, vomiting, headache, ingestion of unpasteurized dairy products, tachycardia, tachypnea, hepatomegaly, splenomegaly, thrombocytopenia, elevated erythrocyte sedimentation rate (esr) (esr), elevated ast and alt, elevated ggt, elevated alkaline phosphatase, elevated alkaline phosphatase/ggt, brucella agglutination titer >160	Brucellosis (with spontaneous splenic rupture)	yes	yes	12	2	2
Acta Medica (Hradec Kralove). 2012;55(3	child nausea vomiting male abdominal pain - right lower back pain rebound tenderness	Acute appendicitis	no	no	1	1	1
Am J Trop Med Hyg. 2013 Jun;88(6):	age greater than 60, elderly, female, farmer, fever, japan, fatigue, eschar at site of arthropod bite, rash on trunk	Malaria - p. vivax(this seems like rickettsial pox)	no	yes	1	>20	16

BMC Infect Dis. 2013 Aug 8;13:369. doi: 10.1186/1471-2334>203-369.	female, young adult, travel to south america, recent travel to southeast asia or latin america, polyarthritis, polyarthritis, contact with monkey, fever,headache, oral ulcers, maculopapular rash .rash, mosquito exposure, mosquito bite,	Mayaro virus infection	yes	no	>20	>20	>20
Int J Surg Case Rep. 2013;4(11):1032-4.	Fever, right lower abdominal pain, vomiting, thrombocytopenia, leukopenia, tachycardia	Dengue fever mimicking acute appendicitis	yes	no	5	4	15
J Infect Dev Ctries. 2014 Feb 13;8(2):237-9. doi:	male, immunocompromised, immunosuppression, india, abdominal pain, diarrhea, fever, positive sirs criteria, fecal leukocytes, positive shigella stool culture, adult (post renal transplant?)	Shigellosis (shigellemia)	no	no	9	3	14
Tex Heart Inst J. 2014 Apr 1;41(2):222-6. doi:	male hypertension adult obese hyperlipidemia smoking history history chest pressure angina syncope st elevation on ecg elevated troponin q waves on ecg	Acute myocardial infarction (heart attack)	no	no	1	1	1
Am J Trop Med Hyg. 2014 Nov;91(5):1035-8. doi: 10.4269/ajtmh.14-	female, thailand, southeast asia, mosquito bite, fever, chills, oral lesions, maculopapular rash, maculopapular rash on trunk spreading to neck and extremities, headache ,conjunctivitis, arthralgia, myalgia, thrombocytopenia,positive dengue serology	Dengue hemorrhagic fever (this case is for zika virus)	yes	yes	5	2	1
Am Fam Physician. 2004 Apr 1;69(7):1727-8.(sinusits)	female adult malaise oliguria caucasian saddle nose deformity glomerulonephritis ear, nose, and throat complaints anca positive sinusitis and renal failure in an elderly patient	Wegener's granulomatosis	yes	no	2	1	1
Swiss Med Wkly. 2007 Jan 13;137(1-2):33.	male adult pharyngitis tonsillitis fever fatigue myalgia lymphopenia thrombocytopenia sore throat oral ulcers positive human immunodeficiency virus (hiv) positive pcr history of unprotected sex	Human immunodeficienc y virus (hiv) positive - initial illness (acute)	no	no	1	1	1
Am J Psychiatry. 2007 Oct;164(10):1491-8.	male elderly parkinsonian symptoms confusion tremor rigidity bradykinesia depression insomnia	Dementia in parkinson's disease (and dementia with lewy bodies?)	no	no	2	1	1
Arthritis Rheum. 2008 Nov;58(11):3632-	male adult gout attacks tophi elevated uric acid obesity hypercholesterolemia swollen joint	Gout	no	no	1	9	1

Emerg Infect Dis. 2010 Feb;16(2):360>20 . doi: 10.3201/eid1602.	male homosexual adult fever sore throat myalgia lymphadenopathy lymphocytosis monocytosis elevated ast and alt	Human immunodeficiency virus (hiv) positive - initial illness (acute) with antiphospholypid syndrome	no	no	1	7	8
Circulation. 2012 Mar 13;125(10):1316-7.	male adult fever back pain tachycardia hypotension positive blood culture leukocytosis valvular vegetations on cardiac echo changing heart murmur	Infective endocarditis	no	no	5	1	1
CMAJ. 2012 Oct 16;184(15):1714. doi: 10.1503/cmaj.111315. Epub 2012	female adult human immunodeficiency virus (hiv) positive steroid use myopathy purple striae lower extremity edema weight gain amenorrhea moon face central obesity hypocortisolismx (ritonavir-fluticasone interaction)	Cushing's syndrome	yes	no	1	1	2
Arthritis Rheum. 2012 Nov;64(11):3759.	female adult polyarthritis positive rheumatoid factor elevated erythrocyte sedimentation rate (esr) (esr) elevated crp xarthritis morning stiffness skin nodules	Rheumatoid arthritis	no	no	9	2	1
Blood. 2012 Jul 26;120(4):708.	female young adult fever myalgia sore throat cervical lymphadenopathy tender cervical lymphadenopathy hepatomegaly splenomegaly lymphocytosis thrombocytopenia atypical lymphocytes	Infectious mononucleosis or ebv infection	no	no	1	6	1
Pediatrics. 2013 Feb;131(2):e616-9. doi:	female adult tachycardia insomnia fatigue anorexia difficulty sleeping weight loss exophthalmos elevated t4	Thyrotoxicosis [hyperthyroidism] (graves' disease)	no	no	1	1	1
Pediatrics. 2013 Jul;132(1):e233-8. doi:	female bradycardia hypotension short stature obese adult dry puffy skin sluggish reflexes elevated tsh level delayed bone age	primary Hypothyroidism (and acute ovarian torsion)	no	no	1	1	1
Intern Med. 2013;52(15):1745-6. Epub	male adult dyspnea elderly syncope deep venous thrombosis	Pulmonary embolism	no	no	1	4	2
BMC Res Notes. 2014 Jan 14;7:36. doi:	female adult dyspnea alterations in blood flow sudden onset shortness of breath prolonged immobilization	Pulmonary embolism	no	no	1	8	1

Indian J Pharmacol. 2014 Jan-Feb;46(1):	initiation of new medication <3 weeks desquamation macules ulcers fever pruritus mucosal ulcers dysphagia dysuria sterile pyuria	Stevens johnson syndrome	yes	no	1	10	2
Cardiovasc J Afr. 2014 Feb 23;25(1): e8-e10.	adult male hypertension diabetes mellitus st elevation on ecg chest pressure angina	Acute myocardial infarction (heart attack)	no	no	1	1	1
Euro Surveill. 2014 Jun 26;19(25). pii:	female adult fever headache cough influenza like symptoms dyspnea chest pain diffuse infiltrates on cxr	Influenza - h5n1 (avian)	no	no	1	14	>20
Br Med J (Clin Res Ed). 1983 Oct 8;287(639	male young adult fever malaise sinus tenderness vision loss pain with palpation of maxillary sinusitis orbital cellulitis periorbital edema	Sinusitis	no	no	1	2	1
J Infect Dis. 1997 Dec;176 Suppl 2:S139-	male adolescent or young adult recent campylobacter infection diarrhea recent infection ascending paralysis weakness acute polyneuropathy	Guillain-barre syndrome	yes	no	1	2	1

Table 3: Dataset 3 - AJTM and OTHER
