# Supplementary material for: An open access medical knowledge base for community driven diagnostic decision support system development
Source: BMC Med Inform Decis Mak. 2019 Apr 27;19:93. doi: 10.1186/s12911-019-0804-1 (PMC6486985; doi:10.1186/s12911-019-0804-1)
Supplement: Supplementary file 4 — Search terms for case retrieval. (PDF 13 kb) [file 12911_2019_804_MOESM4_ESM.pdf]

Appendix B: Search terms for case retrieval

CATEGORY	SEARCH TERMS
FEBRILE SYNDROMES	Fever and Rash
	Fever and Cough
	Fever and Abdominal Pain
	Fever and Headache
	Fever and Joint Pain
	Fever and Diarrhea (non-bloody)
	Fever and Diarrhea (bloody)
	Fever and Dysuria
	Flu-like Illness
	Fever of unknown origin (chronic fever of >3 weeks)
NON-FEBRILE SYNDROMES	Shortness of Breath
	Abdominal Pain
	Cough
	Back pain
	Chest Pain
	Leg Swelling
	Headache
	B-symptoms (weight loss, sweats, etc..)
	Hemoptysis
	Altered mental Status

Table 1: Search terms used to retrieve case for the three evaluation datasets
