# Supplementary material for: An open access medical knowledge base for community driven diagnostic decision support system development
Source: BMC Med Inform Decis Mak. 2019 Apr 27;19:93. doi: 10.1186/s12911-019-0804-1 (PMC6486985; doi:10.1186/s12911-019-0804-1)
Supplement: Supplementary file 5 — Overview of evaluation results. (PDF 79 kb) [file 12911_2019_804_MOESM5_ESM.pdf]

Appendix C: Overview of Evaluation Results

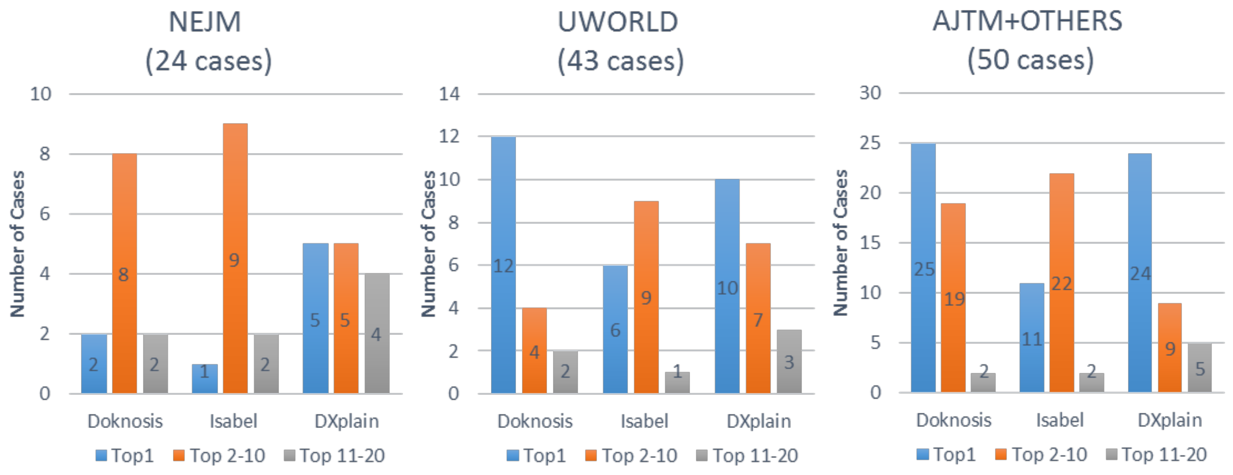

Figure 1: Comparison of Doknosis, Isabel and DXplain analyzing test cases from the three different datasets. Each bar depicts the number of cases for a specific dataset and DDX generator that ranked the correct diagnosis in the reported bucket.
